# Demonstration of a compact plasma accelerator powered by laser-accelerated electron beams

**DOI:** 10.1038/s41467-021-23000-7

**Published:** 2021-05-17

**Authors:** T. Kurz, T. Heinemann, M. F. Gilljohann, Y. Y. Chang, J. P. Couperus Cabadağ, A. Debus, O. Kononenko, R. Pausch, S. Schöbel, R. W. Assmann, M. Bussmann, H. Ding, J. Götzfried, A. Köhler, G. Raj, S. Schindler, K. Steiniger, O. Zarini, S. Corde, A. Döpp, B. Hidding, S. Karsch, U. Schramm, A. Martinez de la Ossa, A. Irman

**Affiliations:** 1 Helmholtz-Zentrum Dresden–Rossendorf, Dresden, Germany; 2 Technische Universität Dresden, Dresden, Germany; 3 Deutsches Elektronen-Synchrotron DESY, Hamburg, Germany; 4 The Cockcroft Institute, Warrington, UK; 5 University of Strathclyde, Glasgow, UK; 6 Ludwig–Maximilians–Universität München, Garching, Germany; 7 Max Planck Institut für Quantenoptik, Garching, Germany; 8 LOA, ENSTA Paris, CNRS, Ecole Polytechnique, Institut Polytechnique de Paris, Palaiseau, France; 9 Center for Advanced Systems Understanding CASUS, Görlitz, Germany

**Keywords:** X-rays, Plasma-based accelerators

## Abstract

Plasma wakefield accelerators are capable of sustaining gigavolt-per-centimeter accelerating fields, surpassing the electric breakdown threshold in state-of-the-art accelerator modules by 3-4 orders of magnitude. Beam-driven wakefields offer particularly attractive conditions for the generation and acceleration of high-quality beams. However, this scheme relies on kilometer-scale accelerators. Here, we report on the demonstration of a millimeter-scale plasma accelerator powered by laser-accelerated electron beams. We showcase the acceleration of electron beams to 128 MeV, consistent with simulations exhibiting accelerating gradients exceeding 100 GV m^−1^. This miniaturized accelerator is further explored by employing a controlled pair of drive and witness electron bunches, where a fraction of the driver energy is transferred to the accelerated witness through the plasma. Such a hybrid approach allows fundamental studies of beam-driven plasma accelerator concepts at widely accessible high-power laser facilities. It is anticipated to provide compact sources of energetic high-brightness electron beams for quality-demanding applications such as free-electron lasers.

## Abstract (web summary)

Particle accelerators based on laser- or electron-driven plasma waves promise compact sources for relativistic electron bunches. Here, Kurz and Heinemann et al. demonstrate a hybrid two-stage configuration, combining the individual features of both accelerating schemes.

## Introduction

In beam-driven plasma wakefield accelerators (PWFAs)^1^, the space charge field of an intense and highly relativistic particle beam propagating through a plasma excites a trailing plasma density wave. Following its driver, the associated plasma wakefield enables the acceleration of a witness electron bunch phase-locked to the accelerating field. For a sufficiently high peak-current drive beam, these plasma wakefields are generated in the blowout regime^2^. In this regime, plasma electrons are completely expelled from the driver vicinity, thereby forming a nearly spherical sheath surrounding an ion cavity^3^. Such a plasma cavity provides extreme accelerating gradients and uniform focusing fields, which are crucial to preserving the witness beam’s emittance—a key quality parameter of particle beams. In addition, PWFAs operating in this regime exhibit a stable wakefield formation, allowing persistent beam-loading conditions to yield witness beams with low-energy spread^4^.

So far, the limited availability of high peak-current drive beams has constrained the development of PWFAs to only a few dedicated radiofrequency (RF) accelerator facilities. Nowadays, compact laser-driven wakefield accelerators (LWFAs)^5^, hosted in many high-power laser facilities worldwide, can deliver GeV-class electron beams^6^ at peak currents exceeding 10 kA^7,8^. In conjunction with an inherently short duration of a few femtoseconds^9^, such LWFA beams constitute ideal drivers for PWFAs^10,11^ at plasma densities above 10^18^ cm^−3^, where accelerating gradients higher than 100 GV m^−1^ can be generated^12^ for the acceleration of ultra-short and ultra-low emittance bunches. Besides, with a sizeable energy spread and emittance, LWFA beams provide attractive attributes for improved resilience to driver instabilities^13,14^. Thus, utilizing LWFA beams as PWFA drivers in a staged LWFA-driven PWFA (LPWFA) combines the unique features of each plasma acceleration method in a compact geometry^15^ with a twofold potential. First, LPWFAs can effectively operate as compact sources of high-brightness electron beams for applications that demand high beam quality^16^. The possibility to excite a strong and stable plasma wakefield using high peak-current LWFA beams enables the implementation of advanced injection schemes, specifically tailored for high-quality witness beam generation based on either selective ionization^17–19^ or plasma-density downramp transitions^20–22^. In addition, the inherent laser-to-beam synchronization offers a key advantage for utilizing auxiliary laser pulses for improved injection control^23^. Second, LPWFAs can serve as a compact development platform to study fundamental PWFA physics complementary to large-scale RF-based PWFA facilities. Among others, extensive studies to improve the driver-to-witness energy transfer efficiency, charge capture efficiency, system stability, emittance and energy spread preservation^24^ can be conducted in widely accessible, high-power laser laboratories. These studies will benefit from the readily available and powerful optical tools^25^ that have already provided the first direct observation of beam-driven plasma wakefields and a first insight into the induced ion motion^26^.

Here we report on two complementary experimental implementations of an LPWFA, performed at 100 TW-class, short-pulse laser facilities: the DRACO laser at the Helmholtz-Zentrum Dresden–Rossendorf (HZDR) and the ATLAS laser at the Ludwig-Maximilians-Universität (LMU) München. In contrast to initial experimental works exploring a transition from laser- to beam-driven modes, which relies on uncontrolled laser pump depletion^27–29^, we employ two separated gas jets individually operating as the LWFA and PWFA stages, respectively. This separation is a fundamental step forward, which allows us to unambiguously demonstrate witness beam acceleration by the evident beam-driven plasma waves, which are exclusively excited by LWFA electron beams. This achievement is a prerequisite to enable the independent control and optimization required to fully explore the aforementioned capabilities of laser-based PWFAs.

## Results

### Drive beam generation

**Fig. 1:**
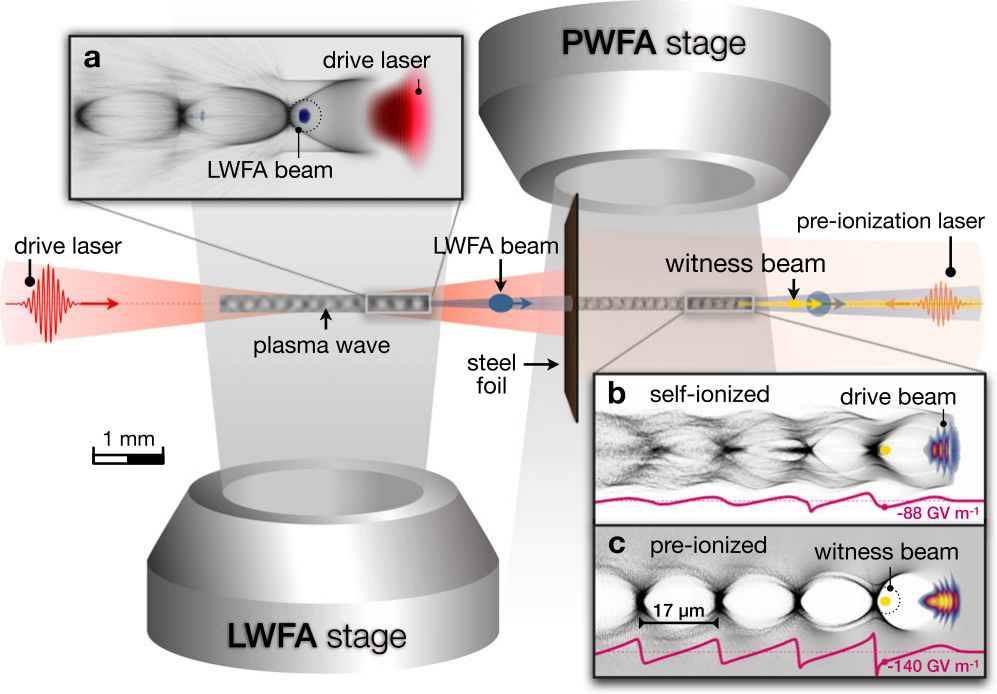
Schematic overview of the experiment. Two consecutive gas jets form the basis of an LWFA-driven PWFA. A high-intensity laser pulse (red) drives an LWFA in the first stage, generating a high peak-current electron beam (blue). The spent LWFA laser is reflected by a thin steel foil, whereas the electron beam propagates into the second stage, acting as the PWFA driver. In the PWFA stage, a witness beam (yellow) is accelerated. In addition, a counter-propagating low-power laser pulse can be applied for generating a pre-formed plasma channel in the PWFA stage prior to the drive beam arrival. **a** Illustration of a plasma wave driven by the high-intensity laser, with electron density in gray. **b** Plasma wave generated by the LWFA electron beam in the self-ionized PWFA regime. **c** Excited plasma wave in the pre-ionized PWFA regime. Images shown in **a**, **b**, **c** are obtained from simulations using the code OSIRIS. The purple line in **b** and **c** represents the longitudinal electric field on axis, considering only the interaction of the drive beam with the second gas jet.

**Fig. 2:**
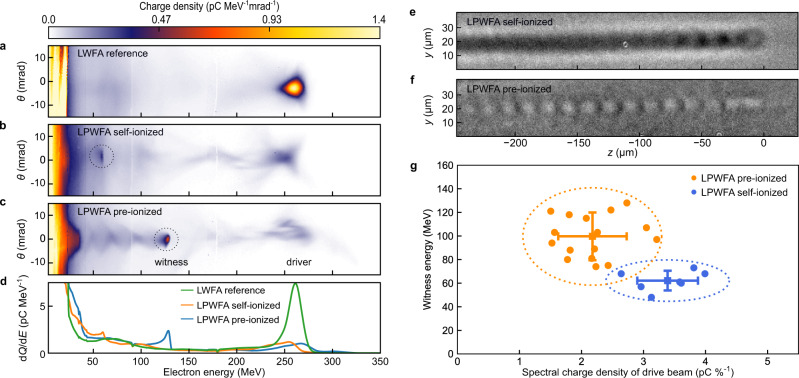
Representative electron spectra, plasma wave shadowgrams, and statistical analysis of witness beam energy. **a** Energy spectrum of LWFA electrons transmitted through the steel foil without operating the PWFA stage, with *θ* representing the divergence. **b** LPWFA spectrum without pre-ionizing laser. **c** LPWFA spectrum with the PWFA stage ionized prior to the drive beam arrival. **d** Charge distribution integrated over ±6 mrad divergence: green line corresponds to **a**, orange to **b**, and blue to **c**. Additional spectra used for determining the statistical parameters can be found in Supplementary Fig. 3, with the measured drive beam parameters from all shots summarized in Supplementary Table 1. **e** Plasma wakefield shadowgram at the center of the PWFA stage, with the drive beam propagating to the right and ionizing the gas by means of its electric field (self-ionized case). The ionized channel (dark region) and several plasma wakefield oscillations directly behind the driver are visible. **f** Corresponding shadowgram of a plasma wakefield in the pre-ionized case. The plasma wave structure is more pronounced and shows more subsequent cavities than in the self-ionized case, indicating a stronger wakefield excitation. **g** Measured witness energy correlated with the remaining spectral charge density of the drive beam. The data points represent spectra exhibiting clear witness beams in the pre-ionized (orange, 37% of shots) and self-ionized case (blue, 28% of shots). The squares denote the mean value of each set with error bars visualizing the root mean square shot-to-shot fluctuations and ellipses encircling the area of 2 SDs. Operating the LPWFA with pre-ionization results in consistently higher witness beam energy gain together with stronger driver degradation.

To optimize the LWFA stage to generate high peak-current drive electron beams, the self-truncated ionization-induced injection scheme^8,30^ is deployed in the first of the two experiments. As sketched in Fig. 1, the LWFA stage consists of a 3 mm-long helium gas jet doped with 3% nitrogen (see “Methods”). The PWFA stage is formed by a 3 mm-long hydrogen gas jet doped with 10% helium, which is located directly behind the first stage, avoiding any vacuum gap in between (see “Methods”). A 12.5 μm-thick steel foil is positioned at the entrance of the PWFA section to reflect the spent laser pulse, whereas the electron beam passes through the foil and drives a purely beam-driven wakefield. In this setup, the PWFA stage can be either self-ionized by the space charge field of the electron drive beam or pre-ionized by a dedicated counter-propagating laser pulse (see “Methods”). A synchronized few-cycle laser pulse provides a side view of the corresponding plasma waves in the PWFA stage via shadowgraphy (see “Methods”). As a reference set, we first recorded shots with only the LWFA stage in operation and the steel foil on position. The resulting electron spectra exhibit typical narrow-band peaks with a 260 ± 9 MeV shot-averaged mean energy ($\bar{E}$), a full-width at half-maximum (FWHM) bandwidth (Δ*E*) of 24 ± 4 MeV, and an FWHM-integrated charge (*Q*) of 104 ± 12 pC. The corresponding shot-averaged spectral charge density, defined as ${\mathcal{S}}=Q/({{\Delta }}E/\bar{E})$, is 11.3 ± 2.1 pC %^−1^. A representative reference spectrum is shown in Fig. 2a. A considerable amount of charge is also observed at low energies up to 30 MeV, attributed to electrons originating from the plasma-density downramp transition at the end of the LWFA stage, consistent with previous measurements^8^. The interaction between the LWFA electrons and the foil increases the divergence of the LWFA beam by 50% (see Supplementary Fig. 1) but does not significantly compromise its ability to drive plasma waves, due to the close proximity between the foil and the PWFA stage.

### Witness beam acceleration

With both jets turned on, the PWFA stage is first operated without pre-ionization. A clear signature of the drive beam interaction with the second stage is observed, as exemplified in Fig. 2b. The shot-averaged spectral charge density decreases to one-third of the value obtained for the LWFA reference shots, due to spectral broadening and charge loss (see Supplementary Table 1 for statistical analysis of 25 consecutive shots), as also reported by Chou et al.^31^. This implies that the drive beam ionizes the ambient gas and transfers a fraction of its energy into the plasma. This hypothesis is confirmed by the shadowgraphy images recorded inside the PWFA stage, depicted in Fig. 2e, which show a narrow plasma filament inside the otherwise neutral gas along the drive beam propagation axis. A few oscillation periods of a plasma wave are observed, clearly demonstrating that the space charge field of the drive beam is sufficiently high to not only ionize the gas but also to excite wakefields. In this self-ionized regime, only a fraction of the drive beam participates in plasma wakefield formation, resulting in a comparatively weak accelerating gradient, as also confirmed by the simulation shown in Fig. 1b. Importantly, distinct signatures of accelerated witness beams are observed in 28% of shots with an average mean energy of 62 ± 4 MeV.

In contrast to the self-ionized regime, creating a pre-formed plasma environment allows the whole drive beam to contribute to the plasma wakefield formation, thus transferring more energy to the plasma and driving a larger amplitude wakefield, as illustrated in simulation Fig. 1c. The associated shadowgram shown in Fig. 2f hence reveals a more pronounced plasma wakefield structure extending beyond ten subsequent cavities, supporting recent observations^26^. This increased interaction with the plasma consequently results in a stronger drive beam degradation (see Supplementary Fig. 4 for supporting simulations). On average (a set of 43 consecutive shots), the spectral charge density of the driver in the pre-ionized condition is reduced to ~17% of the LWFA reference, which indicates that the driver is degraded almost twice as much as in the self-ionized case. As the primary finding, the pre-ionized PWFA section allows the witness beam to gain significantly more energy, as exemplified in Fig. 2c. This correlation between the driver degradation and attainable witness energy is further quantified in Fig. 2g, evaluating only shots that exhibit a clear witness beam signature. Albeit large shot-to-shot fluctuations, a clear differentiation between the self- and the pre-ionized scenarios is observed. In the pre-ionized case, the shot-averaged witness beam energy is substantially increased to 100 ± 5 MeV (37% of shots). As the pre-ionizing laser pulse only influences the second stage behind the foil, where no LWFA laser is present, this increase in witness energy must therefore be attributed to the larger amplitude of the beam-driven plasma wakefield. In agreement with the observation of beam-driven plasma wakefields and the consistent drive beam degradation, this finding provides a conclusive evidence of witness beam acceleration in an LPWFA.

### Acceleration gradient

The experiment delivered a maximum witness energy of 128 MeV, shown in Fig. 2c, which serves as the basis for the following discussion. Considering an acceleration distance of 1.5 mm, from the foil until the end of the plasma density plateau (see “Methods”), and a net energy increase of 66 MeV with respect to the mean value of the self-ionized sample, we thus estimate an effective accelerating gradient ~50 GV m^−1^ higher than in the self-ionized regime. This difference represents a conservative lower limit for the true accelerating gradient, which is independent from the witness initial energy. Such field strength is comparable with those demonstrated in large-scale RF-based PWFA experiments^12,32,33^.

### Witness beam parameters and total energy transfer efficiency

Although the experiment was not optimized to deliver highest beam quality, the witness beam shown in Fig. 2c features almost half the energy spread (6.0%) of the original LWFA drive beam and a similar FWHM divergence (3.8 mrad), albeit at comparatively low charge and approximately half the initial energy of the driver. Nonetheless, we observe a high overall driver-to-witness energy transfer efficiency, defined as the total amount of energy gained by the witness compared to the average initial energy stored in the drive beam (calculated from the pure LWFA reference sample). Taking into account the conservative estimate of the energy gain attributed to the PWFA section (66 MeV) and the measured FWHM-integrated charge of 12 pC, the resulting energy transfer efficiency is 2.9%, which exceeds the value previously achieved in RF-based PWFAs^33^ by about a factor of 5. The parameters of further distinct witness beams are summarized in Supplementary Table 2.

### Particle-in-cell simulation

**Fig. 3:**
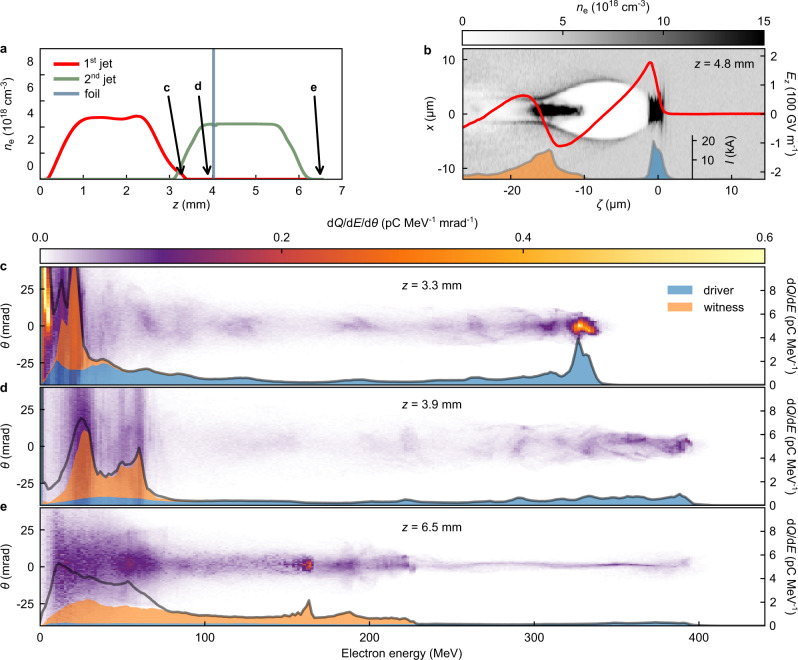
Start-to-end simulation for the pre-ionized case. **a** Plasma density profile in the simulation, according to the actual experimental geometry and measured density profiles for both gas jets. The drive laser pulse propagates to the right. **b** Electron density distribution in the PWFA stage, 0.8 mm after the foil. The LWFA electron beam excites a plasma wakefield, with the witness beam accelerating at the back of the first cavity. *ζ* = *z* − *ct* represents the co-moving coordinate parallel to the drive beam propagation direction *z*, with *c* and *t* denoting the speed of light and time, respectively. The red line is the on-axis longitudinal electric field and the shaded areas on the bottom show the current profiles of the driver (blue) and witness beam (orange). **c** Simulated electron spectrum after the LWFA stage (*z* = 3.3 mm), showing a similar peaked energy distribution of the drive beam (blue) as measured in the experiment. **d** Just before the foil (*z* = 3.9 mm), the wakefield driven by the spent laser pulse leads to an energy gain of the LWFA electrons. **e** After the PWFA stage (*z* = 6.5 mm), a strong degradation of the driver along with energy gain of the witness beam (orange) is observed.

The acceleration process in the pre-ionized scenario can be illustrated with a three-dimensional particle-in-cell (PIC) simulation using the PIConGPU code (see “Methods”). The plasma density profile used in the simulation is shown in Fig. 3a. At the end of the first gas jet, the simulated electron spectrum, depicted in Fig. 3c, shows a narrow-band peak consistent with the experiment when only the LWFA stage is in operation. It also reproduces the generation of a large amount of trailing low-energy background electrons, which are predominantly generated on the first jet exit downramp. At the region in front of the foil, the spent laser pulse is still strong enough to drive a plasma wakefield, which further accelerates the LWFA electrons. The resulting energy gain is visible in the electron spectrum presented in Fig. 3d. After the laser is reflected by the foil, the transition to purely beam-driven acceleration occurs. Figure 3b shows the nonlinear plasma wakefield (red line), driven by the LWFA electron beam (blue shade) during the acceleration of the witness (orange shade). In the simulation, the witness beam is mostly composed of trailing background electrons that went through the foil and get trapped in the beam-driven plasma wake, where they experience peak accelerating fields surpassing 100 GV m^−1^. The resulting broad band energy distribution of the witness after the PWFA section is shown in Fig. 3e. Despite differences in the spectral shape, the simulation renders an acceleration scenario compatible with the measured witness energies. We note that, by neglecting the scattering of the low-energy electrons through the foil, the simulation could have resulted in an overestimation of the fraction of trailing electrons forming the witness beam. Moreover, in the experiment, additional processes may have been at play in the formation of the witness beam, such as electron injection due to a hydrodynamic shock launched from the edge of the foil close to the nozzle.

### Drive-witness bunch pair experiment

**Fig. 4:**
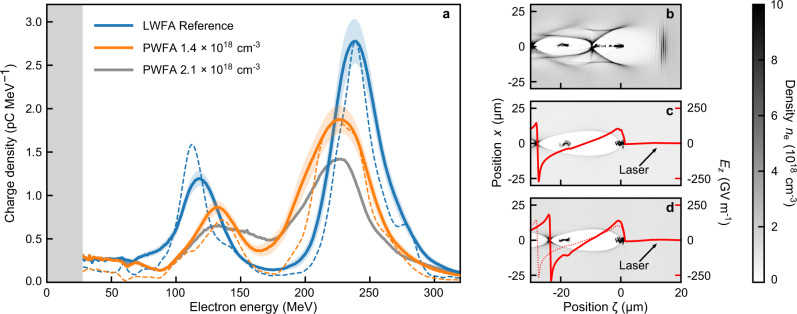
LPWFA using a drive-witness bunch pair. **a** Averaged electron spectra with 95% confidence intervals for LWFA-only reference shots (200 consecutive shots, blue solid line) and with the second stage operated at lower plasma density than the LWFA stage, at 1.4 × 10^18^ cm^−3^ (200 consecutive shots, orange solid line) and 2.1 × 10^18^ cm^−3^ (200 consecutive shots, gray line), respectively. Blue and orange dashed lines correspond to a representative single shot from the associated data set in which a clear distinction of the driver and the witness electron spectrum is even more pronounced. For the higher density PWFA case, a representative single shot cannot be shown due to the onset of spectral overlap between the drive and witness beam, and large shot-to-shot fluctuations. The detection range of the spectrometer starts at 25 MeV. **b** Simulated electron density in the LWFA stage, illustrating that two bunches are injected into the first and second wakefield cavity, subsequently acting as a driver-witness pair in the PWFA stage. *ζ* represents the co-moving coordinate parallel to the laser propagation direction *z*. **c**, **d** Simulation snapshots in the PWFA stage, corresponding to the lower and higher plasma density setting in the experiment, respectively. The red lines correspond to the longitudinal on-axis electric field, showing that the plasma wave is driven by the electron bunch originating from the first LWFA cavity, whereas the contribution of the laser remnant to the wakefield formation is negligible. The accelerating phase witnessed by the electron bunch from the second LWFA cavity is controlled by tuning the plasma density. Further information regarding the pair bunch parameters and simulation details can be found in Supplementary Note 4.

Demonstrating the capabilities of LPWFAs to accelerate witness beams serves as the basis for implementation of various techniques of controlled injection. One key aspect is the ability to insert a controlled pair of electron bunches in close succession into a PWFA stage, where the leading bunch (the driver) excites a plasma wakefield followed by the trailing bunch (the witness) injected at the wakefield-accelerating phase to gain energy^33^. Here, the paired bunch parameters including the inter-bunch temporal separation can be independently characterized and tuned prior to the injection, thus enabling the clear separation between the bunch generation from the acceleration process itself. In the following, we present a proof-of-principle experiment of such a scheme performed in a compact LPWFA module^10^. The bunch pair is generated in the LWFA stage, i.e., a 5 mm-long hydrogen gas jet, by optimizing the shock-front injection technique^34,35^ such that electrons are injected into multiple plasma cavities. The bunch trailing in the second cavity represents the witness for the subsequent PWFA stage, experiencing the wakefield driven by the bunch from the first cavity. This method ensures a defined inter-bunch separation by approximately one plasma wavelength of 17 μm (see Fig. 4b) and a fixed energy ratio with well-separated peaks as observed in the average and single-shot LWFA spectrum (blue solid and dashed lines, respectively) shown in Fig. 4a. To extract the drive-witness bunch pair parameters, a double Gaussian fit was performed on the electron spectrum for each shot (see Supplementary Fig. 7). The average single-shot energy distribution of the driver, i.e., the high-energy distribution, shows a mean energy of 244 ± 1 MeV with an FWHM-integrated charge of 120 ± 5 pC, whereas the witness bunch has approximately half of the energy and a third of the charge, i.e., 119 ± 1 MeV and 36 ± 2 pC, respectively. In this experiment, no laser-blocker foil was used but the distance between the stages was increased, creating a vacuum gap of 6 mm. This way, the laser intensity at the entrance of the PWFA stage, formed by a 1 mm-long hydrogen gas jet, is substantially reduced due to its natural diffraction. This assumption is supported by simulations using the FBPIC code (see “Methods”), which suggest that the laser is already too wide and thus too weak to drive a significant wakefield (see Fig. 4c). Therefore, the laser remnant essentially takes the role of the pre-ionizer for the PWFA stage. In contrast, with a small 1 mrad (FWHM) divergence, the LWFA bunches maintain a high charge density, such that the driver is still able to excite a strong plasma wave in the second stage^26^. To position the witness at the accelerating phase of the beam-driven wakefield, the second stage was operated at a lower plasma density of 1.4 × 10^18^ cm^−3^, approximately one-third of the first stage, creating a plasma wavelength about 1.6 times longer compared to the LWFA stage (see Supplementary Fig. 9). Figure 4a shows averaged and representative single-shot electron spectra for this setting (orange solid and dashed lines, respectively). In accordance with the first experiment, a degradation of the drive bunch charge and energy spread, as well as a ~5% decrease on its mean energy, are observed in both the single-shot and averaged spectra. In turn, the witness bunch is accelerated to 133 ± 1 MeV, representing an energy gain of 14 MeV. Furthermore, comparing the charge contained within the witness bunch before and after the PWFA stage, a charge capture efficiency of close to 70% is estimated, almost one order of magnitude higher than previously achieved in RF-based PWFAs^33^. In this experiment, the overall energy gain of the witness bunch is kept intentionally small to avoid an overlap between the driver and witness spectra, the latter being illustrated in Fig. 4a for an increased density of 2.1 × 10^18^ cm^−3^ (gray line). In this case, the drive bunch degrades further due to a more intense interaction with the plasma, whereas the witness beam is located closer to the end of the cavity and thus experiences a higher accelerating field (Fig. 4d). Consequently, both spectra start to merge. Nevertheless, our results demonstrate the robustness of LPWFAs, which can in principle be scaled to higher energies by increasing the density or length of the second stage, while maintaining a high charge throughput.

## Discussion

The acceleration of witness electron beams is demonstrated in two independent PWFAs driven by intense laser-accelerated electron beams. Successful operation of this scheme therefore substantiates that a variety of PWFA scenarios can be implemented into typical LWFA facilities, which makes PWFA research and applications more accessible. For this purpose, disentangling the PWFA process from the LWFA is a prerequisite to enable the independent control of each acceleration mechanism. Here, such separation has been achieved by both employing a laser-blocker foil or increasing the distance between the two jets. In future implementations, several technical aspects of this staging concept can be improved. Tailored density ramps^36^ and plasma lenses^37^ can be deployed to mitigate emittance growth during transport of the beams over extended distances between the two stages and to facilitate beam matching into the PWFA section. Placing the laser blocker further downstream minimizes beam degradation imposed by the laser–foil interactions^38^. Furthermore, the witness beam energy can be increased by elongating the PWFA stage close to the depletion distance of the driver. The LPWFA platform paves the way for a wide range of hybrid plasma accelerator systems, such as a quality-preserving PWFA energy booster module^10^ based on controlled and tunable drive-witness pair production in the LWFA stage^35^. Ultimately, the high wakefield amplitudes and the inherent laser-to-beam synchronization, unique to the LPWFA scheme, will allow the implementation of advanced internal injection schemes directly inside the PWFA stage, specifically developed for generating ultra high-brightness beams with unprecedentedly low emittance and energy spread^17,18,20^. Therefore, optimized implementations of LPWFAs can be used as beam brightness and energy transformers, delivering high-quality beams at multi-GeV energies, while maintaining a compact setup^15^. Such electron beams would be compliant with beam-quality-demanding light sources such as compact free-electron lasers^16^.

## Methods

The method sections are organized as the following: the first eight sections are dedicated to the first experiment, while the last section corresponds to the second experiment and related simulations.

### Laser system

The high-gradient LPWFA experiment was performed at the DRACO Ti:Sa chirped pulse amplification laser system at the HZDR^39^. The system delivers pulses of 30 fs (FWHM) duration at 800 nm central wavelength. In this work, a pulse energy of 1.7 J was applied on target, after a small energy extraction of about 21 mJ for the counter-propagating pre-ionization laser and the few-cycle probe pulse. The remaining part of the pulse was focused by an *F*/20 off-axis parabolic mirror onto the LWFA stage. The focal spot profile was optimized to a nearly diffraction-limited far field by performing a wavefront correction on the laser near-field with a wavefront sensor (SID4-Phasics) in closed loop with a deformable mirror, resulting in a FWHM spot size of 19.5 μm as measured at the vacuum target focus position. The estimated peak intensity *I*_0_ equals 1.0 × 10^19^ W cm^−2^, corresponding to a normalized vector potential *a*_0_ ≈ 2.1. The spectral shape was measured with spectral-phase interferometry for a direct electric-field reconstruction (SPIDER-A.P.E.) in conjunction with a self-referenced spectral interferometry (WIZZLER-Fastlite). An acousto-optic programmable dispersive filter (DAZZLER-Fastlite) was used in a closed loop for the correction of any dispersion mismatch between the stretcher, compressor, dispersive materials, and beamline optics. During operation, online diagnostics for far-field, near-field, and temporal stability situated at the experimental area were used to ensure stable shot-to-shot performance of the laser.

### Laser-wakefield acceleration stage

In the high-gradient LPWFA experiment, the laser-wakefield acceleration stage was operated in a tailored regime of the self-truncated ionization-induced injection scheme^30^, generating high-charge electron beams. This scheme employs a low ionization threshold (LIT) gas as the plasma medium doped with a small fraction of a high ionization threshold (HIT) gas. The inner electrons of the HIT gas are ionized and subsequently injected only in the vicinity of the intensity peak of the laser pulse located at the front of the plasma bubble. The truncation, which limits the electron injection time, is caused by the nonlinear evolution of the laser pulse in the plasma and the correlated evolution of the plasma cavity^40^. The plasma medium was provided by a 3 mm supersonic de Laval nozzle (Mach 10.4) attached on a fast valve (Parker 9-series) operated using a pre-mixture of helium (97%) and nitrogen (3%) acting as the LIT and the HIT gas species, respectively. Before the experiment, the gas profile was characterized by a dedicated tomographic interferometry setup^41^, yielding a flat top region of ~1.6 mm with density ramps of ~0.6 mm on both sides along the laser propagation axis. The gas pressure was set to 14–16 bar, resulting in a plasma density of 4–4.5 × 10^18^ cm^−3^.

### Plasma-wakefield acceleration stage

For the PWFA stage, a gas nozzle with a geometry identical to the one in the LWFA stage was used, operated with a pre-mixture of hydrogen (90%) and helium (10%) at a plasma density of 3.5–4.2 × 10^18^ cm^−3^, assuming the full ionization of hydrogen and the first level of helium. Such plasma density values were confirmed by evaluating plasma wavelengths recorded on the shadowgraphy images. The use of a gas mixture was intended to, in principle, enable ionization-based injection schemes^18,42^. The PWFA nozzle was oriented at a 90° angle with respect to the LWFA nozzle, to minimize turbulence of jet flow between both stages. The PWFA stage was positioned after the LWFA stage such that the LWFA gas-jet downramp and the PWFA gas-jet upramp were directly adjoined without any vacuum gap in between (see the gas profile in Fig. 3a).

### Laser-blocker foil

A 12.5 μm-thick steel foil was used to reflect the spent LWFA driver laser entering the PWFA stage. Mounted on a rotational disc, the foil was refreshed for each shot. The foil position with respect to both jets could be adjusted. In the work presented here, it was positioned at ~700 μm upstream from the center of the second gas jet. The intensity of the spent LWFA laser at this position is still sufficiently high to induce complex relativistic processes in the foil, which in turn degrade the divergence of passing electron beams^38^. This can be mitigated by increasing the foil distance further downstream of the LWFA stage. Additional beam deterioration imposed by multiple scattering can be minimized by using a thinner low-Z material for the laser blocker and/or by increasing the electron energy.

### Pre-ionizing laser

Optionally, the PWFA stage gas-medium could be ionized prior to the drive beam arrival. This is achieved by a ~20 mJ laser counter-propagating under a shallow angle through the PWFA stage, about 1 ps before the arrival of the LWFA beam. A curved mirror with a focal length of *f* = 1 m was used to focus the laser to a spot size of ~120 μm (FWHM) corresponding to a focal peak intensity of 4 × 10^15^ W cm^−2^, which is well above the ionization threshold for hydrogen and helium. The ionization laser is prevented from entering the LWFA stage by the steel foil. However, the intensity of the pre-ionization laser was sufficiently low to not compromise the integrity of the blocker foil.

### Few-cycle probe laser

A few-cycle laser pulse was used for ultrafast probing of the PWFA stage by recording shadowgrams of the plasma waves. The probe generation setup consists of a 1.0 m-long hollow core fiber filled with 2.0 bar of neon, which was seeded using a 1 mJ beam picked up from the main laser pulse, thus inherently synchronized. After this laser pulse was spectrally broadened inside the fiber, it was compressed using chirped mirrors to a pulse length of 9.2 fs measured by a spectral-phase interferometry for a direct electric-field reconstruction (SPIDER-A.P.E). The output energy was measured to be 0.4 mJ at a beam diameter of 7 mm. This probe beam was directed to the center of the PWFA stage, transversely illuminating the plasma wakefield, which was imaged by a long working distance objective onto a 14-bit charge-coupled device (CCD) camera. This created shadowgram has a spatial resolution of 0.46 μm per pixel. The gray scale in the shadowgraphy images cannot be directly compared to each other and does not represent the absolute plasma electron density. This is because during the post processing of the raw images, the absolute value of the pixel count is adjusted to improve the image contrast.

### Electron beam characterization

The electron beam spectral distributions were determined using a 0.4 m-long permanent magnet dispersive dipole spectrometer with a magnetic field strength of 0.9 T. Phosphor-based scintillating screens (Konica Minolta OG 400), imaged to 12-bit CCD cameras, were positioned such that the energy resolution is optimized with point-to-point imaging up to 200 MeV. At higher energies, the readout error is dominated by the beam pointing error, with a readout uncertainty of −1.2/+1.6% at 300 MeV and −2.5/+3.1% at 400 MeV for a 6 mrad pointing error^39^. The overall detection range is 2–550 MeV. To deduce the beam charge-energy distribution, the absolute-charge response of scintillating screens was calibrated against the Electron Linac for beams with high Brilliance and low Emittance accelerator in a separate campaign^43^.

### Particle-in-cell simulations

The three-dimensional start-to-end simulation shown in Fig. 3 was performed with the PIC code PIConGPU^44,45^, version 0.4.2^46^. The simulation closely approximates the experimental parameters of the pre-ionized LPWFA by modeling the measured transverse laser pulse profile by including higher Laguerre-Gauss laser modes, as well as modeling the measured gas density and gas mixture used in both the LWFA and PWFA stages. As in the experiment, a foil is inserted after the PWFA upramp to reflect the laser. For this purpose, the simulated foil was implemented with 50 times the critical density, which is sufficiently dense to lead to the laser-plasma mirror effect. This approach neglects density perturbations around the foil and possibly underestimates the divergence increase due to fields within the foil, as observed in the experiments and also reported in a dedicated study^38^. Closely mimicking the experiment, the central wavelength of the laser is 800 nm, the total energy 1.4 J, the pulse duration 30 fs, and the spot size 19 μm (both FWHM intensity). The moving-window frame of the simulation has a total size of 768 × 4608 × 768 cells and propagates for 300,000 iterations. The spatial resolution is 177 × 44.3 × 177 nm with a temporal resolution of 72.8 as. The electromagnetic field evolution is simulated with the Lehe solver^47^ including a binomial filter^48^, whereas the particle motion is computed using the Boris pusher^49^. Particles influence the fields via the Esirkepov current deposition scheme^50^ with a triangular-shaped density cloud macro-particle shape^51^. Ionization was treated via a combined BSI^52^ and ADK^53^ model. Simulations used for the illustration of the concept in Fig. 1 were performed using the code OSIRIS^54^.

### Drive-witness bunch pair experiment

The driver-witness pair LPWFA experiment was performed at the ATLAS Ti:Sa laser system at LMU München, using a pulse energy of 2.5 J on target with a FWHM duration of 28 fs (80 TW) at 800 nm central wavelength. The pulses were focused by an *F*/25 off-axis parabola to a spot size of ≈24 μm FWHM. The corresponding peak intensity is determined by measurements of the on-target laser energy, the pulse duration, and a high-contrast focal spot analysis to 6.8 ± 0.5 × 10^18^ W cm^−2^ and *a*_0_ ≈ 1.8.

The target consists of two de Laval-type nozzles providing supersonic, pure hydrogen gas jets for the LWFA and PWFA stage. The flow in the LWFA stage with an outlet diameter of 5 mm and a Mach number of 6.35 is partially obstructed by the sharp edge of a silicon wafer, leading to the formation of a supersonic shock^34^. The plateau density after the shock is determined to 3.7 × 10^18^ cm^−3^ by in situ interferometry and the full longitudinal density profile is given in Supplementary Fig. 5. The shock leads to the generation of a dual-energy bunch pair, injected into the first and second period of the plasma wakefield. These two bunches, forming the driver and witness in the subsequent PWFA stage, are hence separated by approximately one plasma wavelength *λ*_*p*_ ≃ 17 μm.

The PWFA stage consists of a 1 mm diameter Mach 5 nozzle with a peak density of 1.4 × 10^18^ cm^−3^. The nozzle is placed downstream of the first stage with a vacuum gap of ≈5.5–6 mm. As no laser blocker is used, the gap ensures that diffraction reduces the laser intensity to below 1 × 10^17^ W cm^−2^, such that the laser only acts as a pre-ionizer for the PWFA stage.

A permanent magnet electron spectrometer of the same magnet design and strength as that at HZDR, but a length of 0.8 m, is used for electron detection, in combination with an absolutely calibrated CAWO OG16 phosphor screen^43^. The screen is placed parallel to the magnet edge and imaged with 14-bit CCD cameras, measuring energies starting from 25 MeV. Its accuracy was enhanced by placing a second CAWO OG16 screen (“pointing screen”) at the spectrometer entrance to reference the electron beam pointing for every shot.

The simulations shown in Fig. 4b–d and in Supplementary Fig. 9 were performed using the code FBPIC^55^, separately for the LWFA and electron transport together with PWFA stages, to efficiently use the computational resources. At the interface between both simulation domains, the simulated electron bunch divergence is numerically adjusted to the measured value. The first simulation yields the dual-bunch charge and longitudinal distribution, as well as the laser parameters at the exit of the LWFA stage. A box size of 70 μm in length with 80 μm radius, with 2500 longitudinal and 500 radial grid points, and 2 azimuthal modes is used, corresponding to a cell resolution of 28 nm × 160 nm, respectively. Each cell is filled with 16 macroparticles. The driving laser pulse is initialized with a pulse duration of 30 fs (FWHM) and a focal spot size of *w*_0_ = 20 μm (FWHM). This small discrepancy to the experiment is required to obtain matching charge and energy figures, but should not influence the laser divergence after the target, because the laser propagation in the target is dominated by self-focusing. The modeled gas density is a piecewise linear approximation of the measured profile. The second simulation takes the output from the first, corrected for the experimental electron bunch divergence, and propagates the bunches through the vacuum gap and PWFA stage with a 65 μm-long and 160 μm-radius box, divided into 32.5 nm × 213 nm cells with 16 particles and 4 azimuthal modes.

## Supplementary information

Supplementary Information

## Data Availability

The input data set for the numerical simulation visualized in Fig. 3 is available online^56^. The data that support the figures and further findings of this article are available from the corresponding authors upon reasonable request.
