# Supplementary material for: Demonstration of a compact plasma accelerator powered by laser-accelerated electron beams
Source: Nat Commun. 2021 May 17;12:2895. doi: 10.1038/s41467-021-23000-7 (PMC8129089; doi:10.1038/s41467-021-23000-7)
Supplement: Supplementary file 1 — Supplementary Information [file 41467_2021_23000_MOESM1_ESM.pdf]

Supplementary Information
Demonstration of a compact plasma accelerator powered by laser-accelerated electron beams

T. Kurz,[1,2,*] T. Heinemann,[3,4,5,*] M. F. Gilljohann,[6,7] Y. Y. Chang,[1] J. P. Couperus Cabadağ,[1] A. Debus,[1] O. Kononenko,[8] R. Pausch,[1] S. Schöbel,[1,2] R. W. Assmann,[3] M. Bussmann,[1,9] H. Ding,[6,7] J. Götzfried,[6,7] A. Köhler,[1] G. Raj,[8] S. Schindler,[6,7] K. Steiniger,[1] O. Zarini,[1] S. Corde,[8] A. Döpp,[6,7] B. Hidding,[4,5] S. Karsch,[6,7] U. Schramm,[1,2] A. Martinez de la Ossa,[3] and A. Irman[1]

[1]*Helmholtz-Zentrum Dresden–Rossendorf, Bautzner Landstrasse 400, 01328 Dresden, Germany*
[2]*Technische Universität Dresden, 01062 Dresden, Germany*
[3]*Deutsches Elektronen-Synchrotron DESY, Notkestraße 85, 22607 Hamburg, Germany*
[4]*The Cockcroft Institute, Keckwick Lane, Warrington WA4 4AD, United Kingdom*
[5]*University of Strathclyde, 107 Rottenrow, Glasgow G4 0NG, United Kingdom*
[6]*Ludwig–Maximilians–Universität München, Am Coulombwall 1, 85748 Garching, Germany*
[7]*Max Planck Institut für Quantenoptik, Hans-Kopfermann-Strasse 1, 85748 Garching , Germany*
[8]*LOA, ENSTA Paris, CNRS, Ecole Polytechnique, Institut Polytechnique de Paris, 91762 Palaiseau, France*
[9]*Center for Advanced Systems Understanding CASUS, 02826 Görlitz, Germany*

This supplementary document provides additional information on the two experimental configurations presented in the main manuscript. Additional experimental data, simulations and discussions regarding the high-gradient LPWFA experiment are presented in Supplementary Figures 1-4, Supplementary Tables 1-2 and Supplementary Notes 1-3. Supplementary experimental data, simulations and discussions related to the drive-witness bunch pair experiment are presented in Supplementary Figures 5-9, Supplementary Table 3 and Supplementary Note 4.

* These authors contributed equally to this work

Supplementary Figures

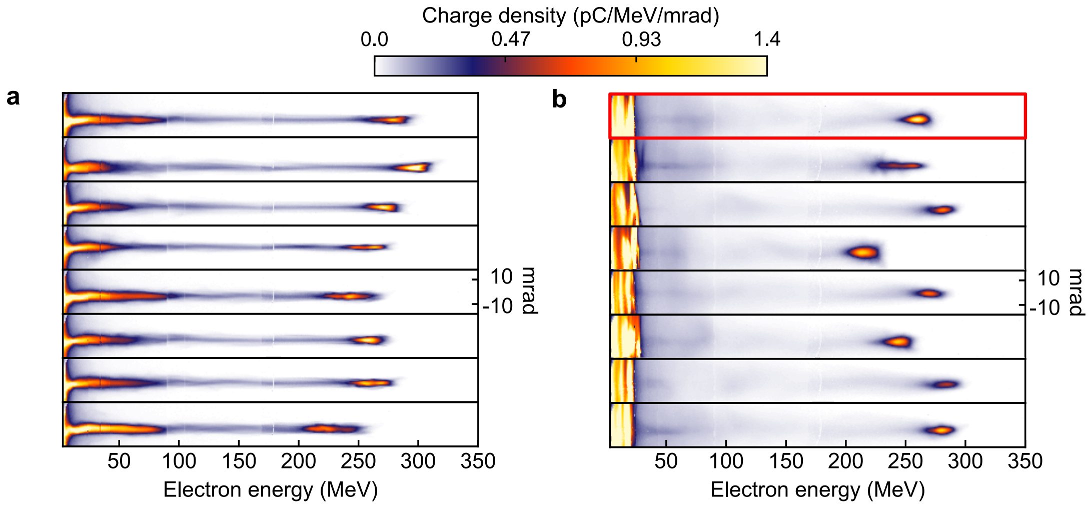

Supplementary Figure 1. **LWFA electron spectra with and w/o foil**. **a** Eight consecutive energy spectra characterize the LWFA performance without the presence of the laser blocker foil. The mean divergence (FWHM) of the high-energy peak is measured as 4.2 ± 0.2 mrad. **b** Eight consecutive electron spectra recorded when the foil was placed on the laser axis. This experimental condition is referred to as *LWFA reference* in the main manuscript, where the spectrum highlighted by the red frame is shown in Fig. 2a. The propagation of the drive beam through the foil results in a ∼50 % increase in mean FWHM–divergence, but does not affect other beam parameters.

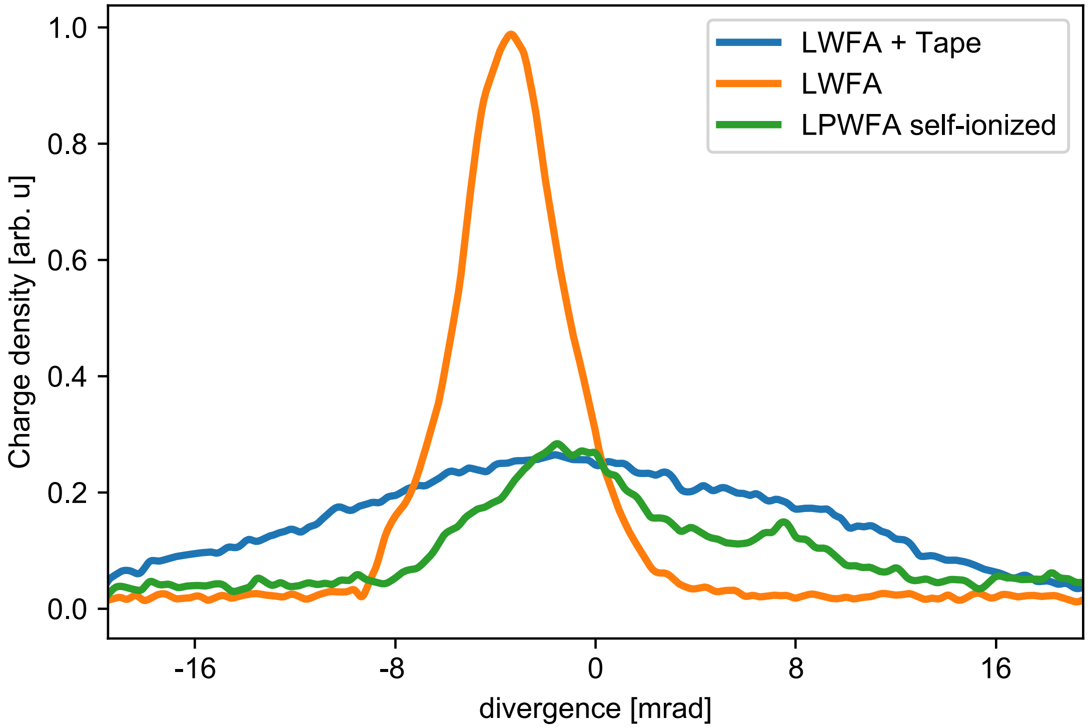

Supplementary Figure 2. **Normalized charge density along the transverse electron distribution.** The lines represent energy–slices at ∼150 MeV detected on the electron spectrometer. The initial LWFA output (orange line) shows a high charge density peak on axis with a divergence of 5.2 mrad. The interaction with the foil (blue line) broadens this peak and increases the divergence to 24.3 mrad. Activating the PWFA stage (green line) reduces the overall charge, but the interaction with the plasma keeps the charge density on axis at the same level as in the case without the PWFA stage, which reduces the divergence to 16.0 mrad.

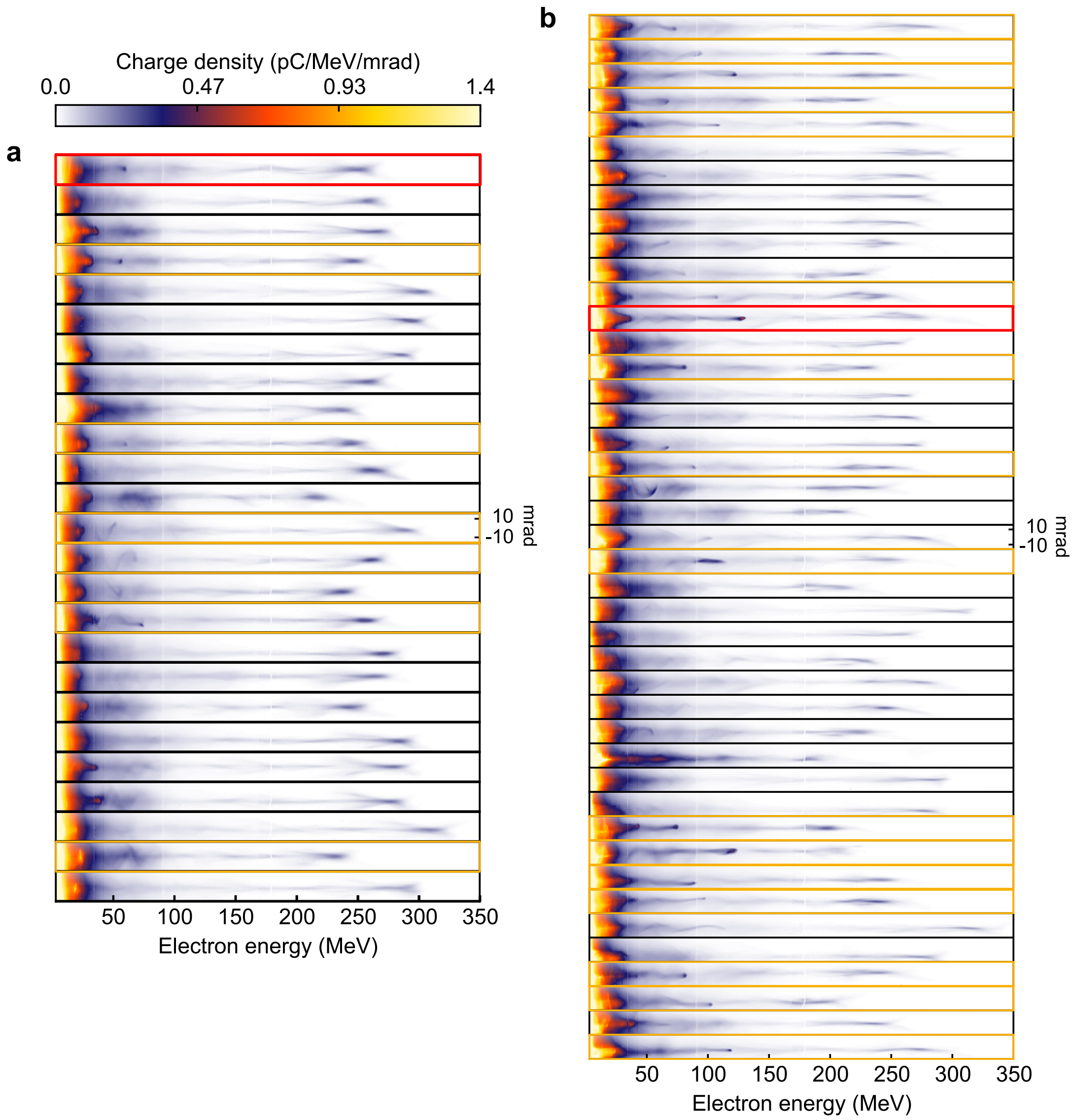

Supplementary Figure 3. **Recorded LPWFA electron spectra.** The spectra shown in **a** represent the LPWFA operated without a pre–formed plasma environment and **b** shows the spectra with the PWFA section ionized prior to the arrival of the drive beam. The red and yellow frames indicate the spectra presented in Fig. 2b-c and Fig. 2g in the main manuscript, respectively.

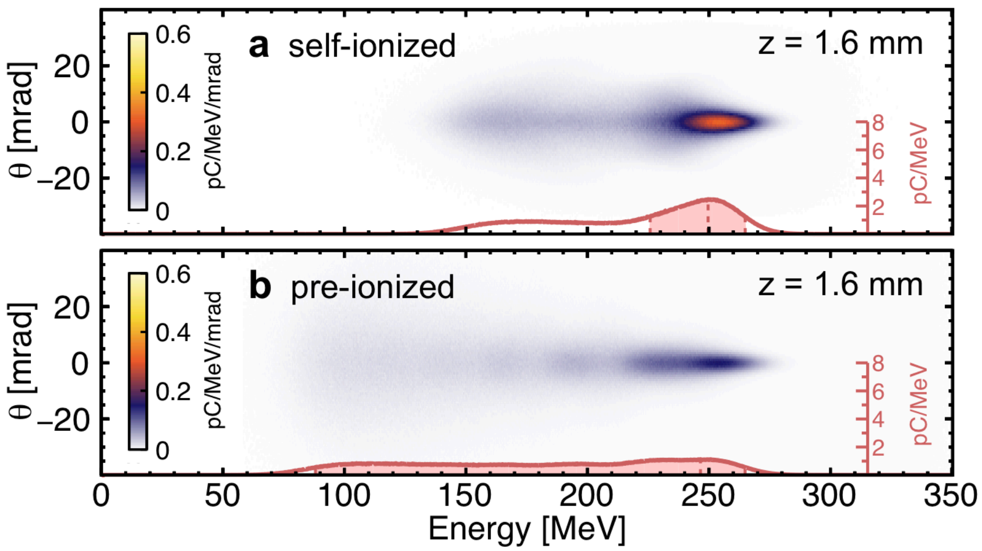

Supplementary Figure 4. **LPWFA drive beam deceleration simulation results.** Simulated drive beam spectra after the propagation of 1.6 mm with respect to the start of the PWFA density plateau. The drive beam distribution is visualized by a 'virtual' 2D spectrometer image. The inset y-axis (red scale) shows the angular integral of the charge density (pC/MeV). **a** Shows the drive beam in the self–ionized LPWFA case. **b** Depicts the drive beam in the pre–ionized LPWFA case. Similar to the experimental findings, it is observed that the energy spread of the drive beam in the pre–ionized case is significantly enlarged and thus its spectral charge density is reduced.

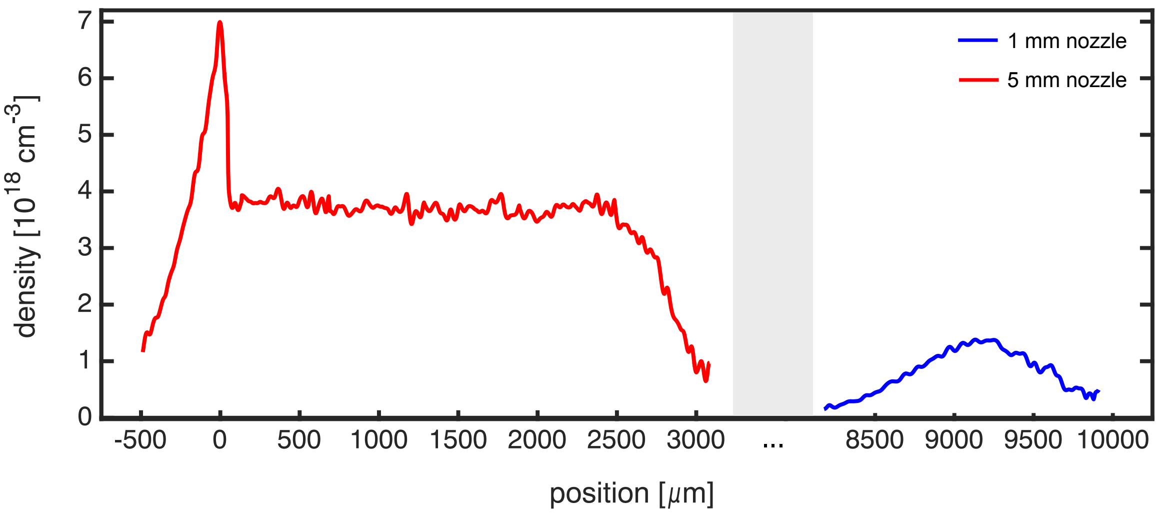

Supplementary Figure 5. **Electron density profile of the drive-witness bunch pair LPWFA experiment.** The lines represent the plasma density profile of the first 5 mm (red) and second 1 mm (blue) gas jet, along the axis of interaction. The high density peak on the left is created by the supersonic shock.

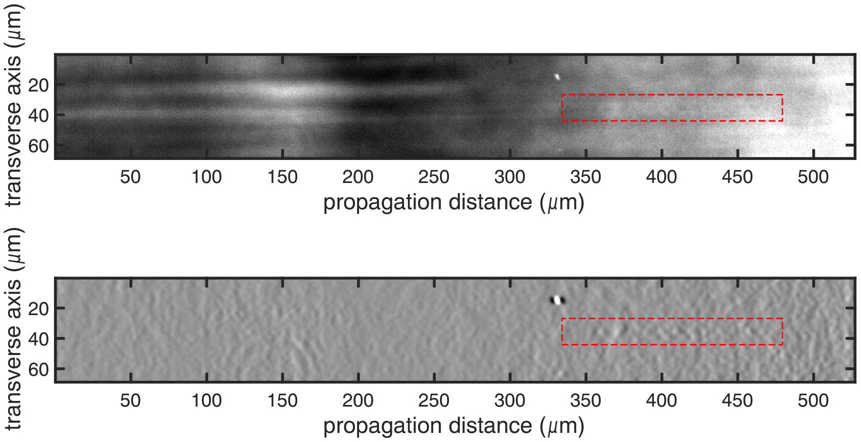

Supplementary Figure 6. **Beam-driven plasma wave shadowgram inside the PWFA stage from the driver-witness bunch pair experiment.** Top: Raw image of plasma wave in the second jet. The red rectangle highlights the region of the modulation. Bottom: Processed image of plasma wave. A 20-pixel moving average of each horizontal line of the raw image is subtracted in order to reduce the horizontal striations caused by the ionization front of the laser (c.f. Gilljohann et al., PRX 9, 011046 (2019). This treatment highlights high-frequency modulations along the propagation axis.

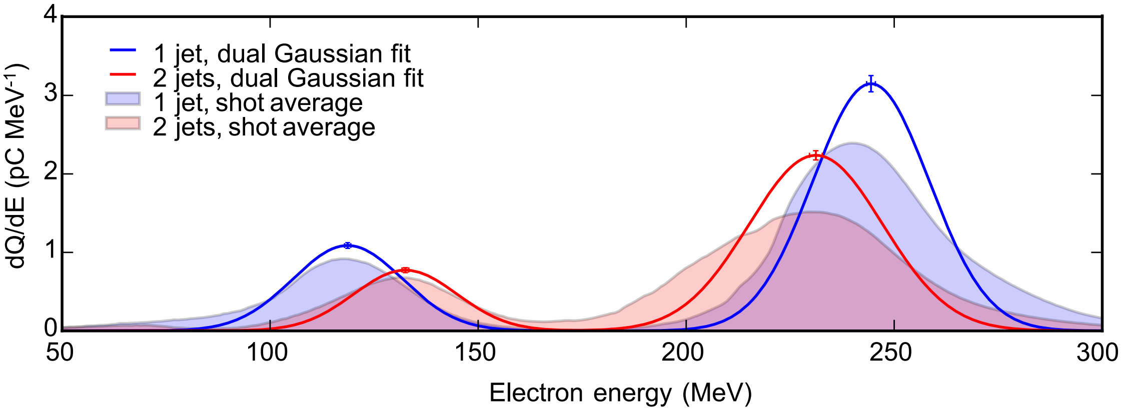

Supplementary Figure 7. **Average drive-witness pair spectra and peak analysis.** Blue shaded area: 197-shot-averaged LWFA reference spectra, showing a dual-energy bunch distribution. The drive bunch possesses a higher energy than the witness. To identify the energy peaks of such a bunch pair, a double Gaussian fit was performed on the electron spectrum for each shot. The solid blue line represents the double Gaussian with the shot-averaged fit parameters for the reference set. Red shaded area: 165-shot-averaged electron spectra with both the LWFA and PWFA stages turned on. The solid red line represents the corresponding double Gaussian fit. The error bars indicate the standard error of the average peak position. Note that the deviation of the fits from the measured data is mainly caused by the broadening of the average spectra due to shot-to-shot fluctuations. A systematic deceleration of the drive beam and an acceleration of the witness is clearly observed.

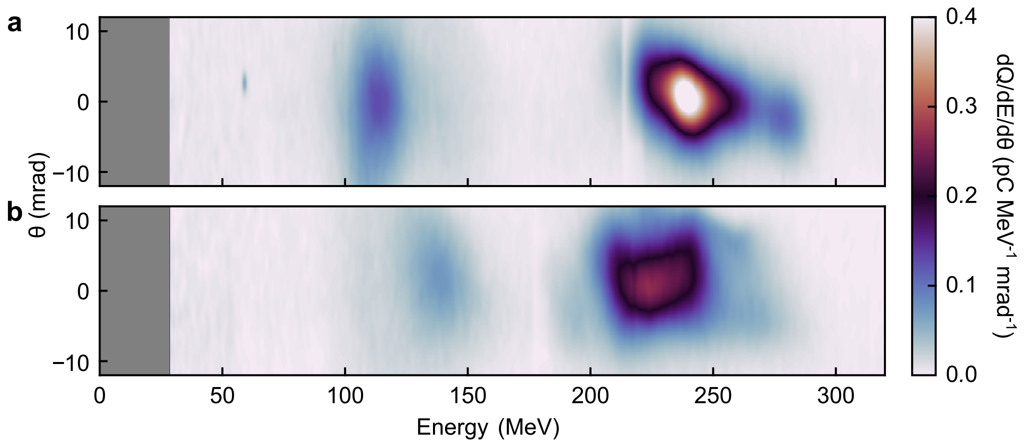

Supplementary Figure 8. **Representative electron spectra in the drive–witness bunch pair experiment.** **a** LWFA-only reference spectrum showing the drive bunch peak at ∼240 MeV and the witness at ∼120 MeV. **b** LPWFA spectrum presenting the driver deceleration to ∼230 MeV and witness acceleration to ∼140 MeV. The associated charge-density profiles are presented in Fig. 4a of the manuscript. Note that the divergence axis only refers to the apparent divergence behind the pointing screen.

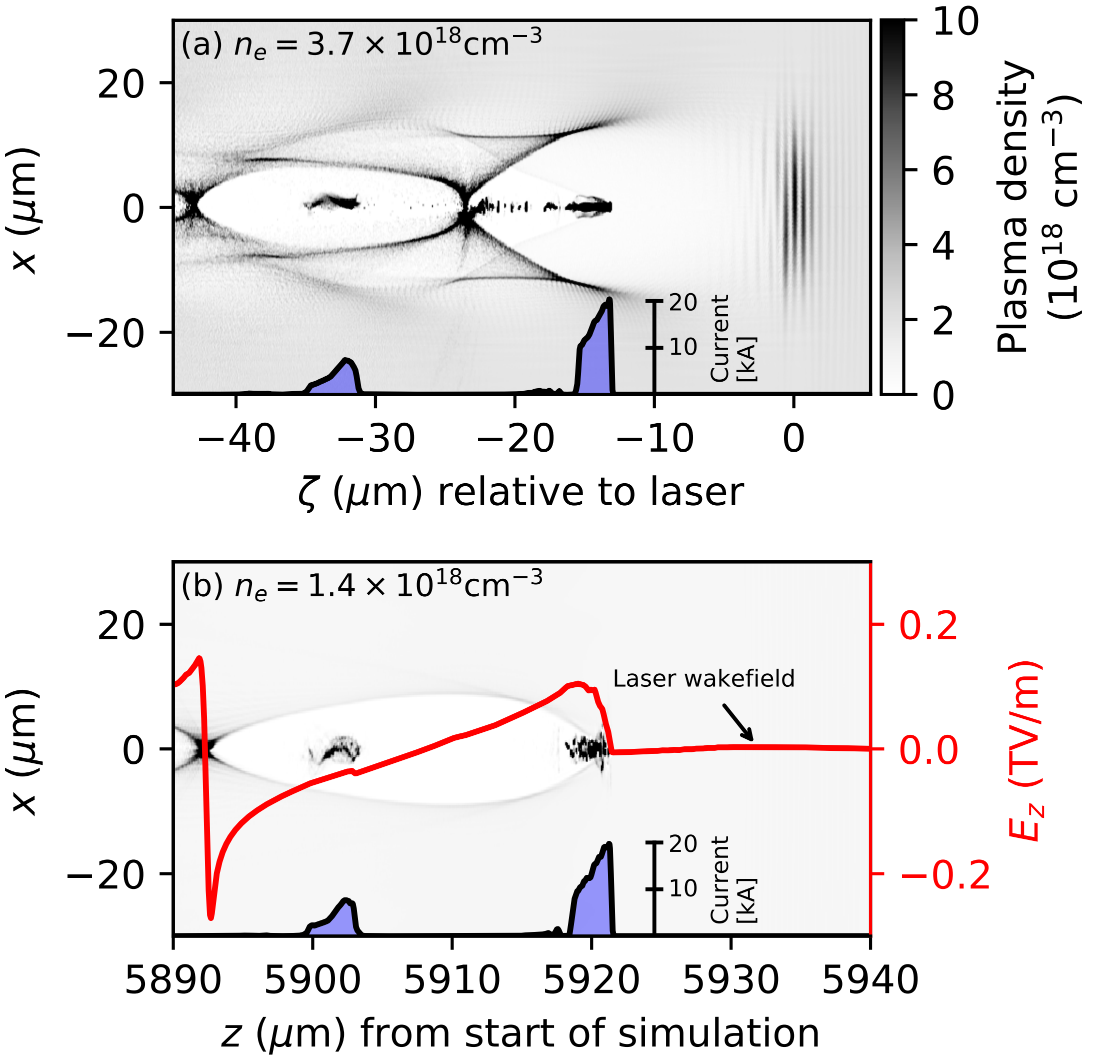

Supplementary Figure 9. **Numerical simulation of the drive–witness bunch pair experiment.** Quasi-3D simulation using FBPIC [3] of the LWFA and PWFA stage. **a** In the LWFA stage, two individual bunches have been injected by the shock into the first and second bucket, respectively. These bunches form the driver and witness in the PWFA stage. **b** The simulation shows that the laser, located approximately 12 µm in front of the drive bunch, has sufficiently diffracted in the vacuum gap to only drive a negligible plasma wave, as visible by the absence of any strong field gradient before the driver bunch. Note that the witness bunch, while being close to the wakefield zero crossing, still experiences an accelerating field, which is consistent with the energy gain observed in the experiment. This figure is also illustrated in the main text as Fig. 4b-c.

Supplementary Tables

Supplementary Table 1. **Drive beam parameters in the high-gradient LPWFA experiment.** The parameters represent the three experimental sets discussed in the main manuscript, the LWFA reference, self–ionized LPWFA and pre–ionized LPWFA. All quantities are evaluated within the FWHM-region of the driver energy peak and averaged over all shots shown in Supplementary Fig. 1b and Supplementary Fig. 3, respectively. The LWFA reference set contains 8 shots, the self–ionized LPWFA set 25 shots and the pre–ionized LPWFA set 43 shots. The error given for each value denotes the standard error of the mean.

	LWFA reference	LPWFA self–ionized	LPWFA pre–ionized
Q [pC]	104 ± 12	48 ± 3	40 ± 2
ΔE [MeV]	24 ± 4	36 ± 2	51 ± 3
$\bar{E}$ [MeV]	260 ± 9	260 ± 5	235 ± 5
$\mathcal{S}$ [pC/%]	11.3 ± 2.1	3.5 ± 0.3	1.9 ± 0.2

Supplementary Table 2. **Witness beam parameters in the high-gradient LPWFA experiment.** The parameters of the five most prominent witness beams observed in the pre–ionized regime are shown. Shot #3 (bold column) is visualized in the main text in Fig. 2c. All witness beam parameters are deduced after subtracting the background formed by decelerated drive beam electrons.

Shot	#1	#2	**#3**	#4	#5
Q_Δ [pC]	4.4	1.6	**12.0**	19.8	3.4
ΔE [MeV]	4.8	4.1	**7.8**	12.8	5.3
$\bar{E}_\Delta$ [MeV]	122	108	**128**	119	118
$\Delta E/\bar{E}$ [%]	4.0	3.8	**6.0**	10.7	4.5
$\mathcal{S}_\Delta$ [pC/%]	1.1	0.4	**2.0**	1.9	0.7
θ_{FWHM} [mrad]	5.4	2.9	**3.8**	5.9	3.5

Supplementary Table 3. **Beam parameters measured in the drive-witness bunch pair LPWFA experiment**. Mean energy $\bar{E}$, FWHM bandwidth ΔE, charge Q and peak charge density $d(Q/E)$ of the drive and witness bunches as derived from Gaussian fits to the corresponding spectral peaks. Numbers are given both for the reference set with only one jet (LWFA-case, average over 197 shots) and for the two-jet case (LPWFA, averaged over 165 shots). The entire dataset contains 200 shots for both cases. For analysis 3 and 35 shots, respectively, were excluded due to pointing issues and spectral overlap of both bunches. Columns labeled by "s.d." give the rms shot-to-shot fluctuation of the respective quantity.

	LWFA	LWFA s.d.	LPWFA	LPWFA s.d.
**Driver**				
$\bar{E}_\mathrm{d}$ [MeV]	244 ± 1	15	231 ± 2	20
ΔE_d [MeV]	36 ± 1	11	39 ± 1	11
Q_d [pC]	120 ± 5	67	94 ± 4	40
$d\,(Q/E)_\mathrm{d,max}$ [pC/MeV]	3.1 ± 0.2	1.5	2.2 ± 0.1	0.8
**Witness**				
$\bar{E}_\mathrm{w}$ [MeV]	119 ± 1	11	133 ± 1	11
ΔE_w [MeV]	31 ± 1	7	31 ± 1	7
Q_w [pC]	36 ± 2	19	25 ± 2	14
$d\,(Q/E)_\mathrm{w,max}$ [pC/MeV]	1.1 ± 0.1	0.5	0.8 ± 0.1	0.4

Supplementary Notes

Supplementary Note 1: High-gradient LPWFA experiment – supplementary experimental data

In the high-gradient LPWFA experiment, the LWFA stage is first optimized for generating high peak-current electron beams which serve as drivers of the PWFA stage. The LWFA electron beams are then characterized with and without introducing the 12.5 µm thick laser blocker foil, as shown in Supplementary Fig. 1. Eight consecutive electron spectra recorded without the foil are presented in Supplementary Fig. 1a, showing consistent narrow-band high-energy peaks with an average full width at half maximum (FWHM) divergence of 4.2 ± 0.2 mrad. In addition to the high-energy peaks, the spectra show a broadband electron background with charge and divergence increasing towards lower energies. With the laser blocker foil in place, the distribution of these background electrons is altered significantly. The eight consecutive shots presented in Supplementary Fig. 1b show an overall dilution of the broadband background and a highly diverging low-energy distribution extending up to 30 MeV. Importantly, the mean energy, energy spread and charge of the drive beam are preserved. After the passage through such a thin foil, only the divergence of the beam is increased to 6.2 ± 0.4 mrad, reducing the number of electrons per mrad. The divergence increase is generally more severe for lower electron energies. However, the integrated charge over the whole detection angle is conserved, as in the LWFA reference shots without the foil.
To illustrate this characteristic, the normalized charge density along the transverse electron distribution at an energy slice of ∼150 MeV is depicted in Supplementary Fig. 2 for three electron spectra representing different experimental conditions. In the pure LWFA configuration (orange line), a narrow and prominent peak with a divergence of 5.2 mrad (FWHM) is observed. Integrating the charge detected on the spectrometer between 100–200 MeV for the LWFA data set (see Supplementary Figure 1a leads to an average of 99 ± 6 pC. Inserting the laser blocker foil (blue line) significantly broadens the electron distribution yielding an increased divergence of 24.3 mrad, whereas the integrated charge remains almost constant at 95 ± 4 pC. When the PWFA stage is turned on (green line), the interaction leads to an overall reduction of the observed charge to 54 ± 7 pC. In particular the amount of "off-axis" electrons decrease significantly while the plasma lens effect of the second jet hinders the "on-axis" electrons to diverge, keeping their quantity about the same as for the LWFA shots with foil. The FWHM-divergence is then reduced to 16.0 mrad. Since the PWFA section starts directly behind the foil, the initial spot size of the driver remains unchanged and its further evolution is governed by the plasma interaction. Therefore, employing the laser blocker foil allows to unambiguously isolate the beam-driven from the laser-driven acceleration, without compromising the capability of the LWFA electron beam to drive a PWFA.
In order to substantiate that the observed witness beams are accelerated in a purely beam-driven wakefield, the LPWFA is operated with and without a pre–formed plasma environment in the PWFA section. In total 68 electron spectra are recorded for these experimental conditions, with the pre–ionization laser being repeatedly switched on and off to confirm its effect on the witness beam acceleration behaviour. All individual spectra can be found in Supplementary Fig. 3, where the 25 shots without the pre–formed plasma are presented in Supplementary Fig. 3a and the 43 shots with pre–ionization shown in Supplementary Fig. 3b. In comparison to the LWFA reference, the pre–ionized LPWFA spectra systematically reveal a substantially stronger drive beam degradation than the self–ionized LPWFA.

Supplementary Note 2: High-gradient LPWFA experiment – witness beam parameters & calculation of total energy transfer efficiency

The average drive beam parameters discussed in the main manuscript are derived taking into account all presented spectra and are summarized in Supplementary Table 1. Here, ΔE symbolizes the FWHM–energy spread of the drive beam high-energy peak. Furthermore, Q denotes the drive beam charge, $\bar{E}$ the mean energy and $\mathcal{S}$ the spectral charge density, with all values evaluated in this FWHM–region. Even though this experiment was not yet optimized for generating highest-quality witness beams, the achieved witness beam parameters already emphasize the potential of LPWFA systems to ultimately deliver beams with largely improved quality features compared to the initial LWFA output. For instance, these witness beams already exhibit promising features such as a significantly lowered energy spread $\Delta E/\bar{E}$ and a similar FWHM-divergence θ_{FWHM} at half the energy as compared to the initial LWFA performance (without foil interaction) with an energy spread of 10.4 %. The parameters of the five most prominent witness beams are summarized in Supplementary Table 2.
The analysis of the witness parameters allows for an estimation of the driver–to–witness energy transfer efficiency R_{eff}. For this, we compare the total energy of the drive beam from the LWFA reference configuration to the total amount of witness beam energy gain solely attributed to PWFA. The latter can be deduced by subtracting the average witness

beam energy in the self–ionized case ($62 \pm 4\,\mathrm{MeV}$) from the maximum achieved in the pre–ionized case ($128\,\mathrm{MeV}$). This yields a conservative lower limit on the efficiency of

$$R_{\mathrm{eff}} = \frac{\bar{E}_w Q_w}{\bar{E}_d Q_d} \approx \frac{66\,\mathrm{MeV} \cdot 12\,\mathrm{pC}}{260\,\mathrm{MeV} \cdot 104\,\mathrm{pC}} = 2.9\,\%.$$

Supplementary Note 3: High-gradient LPWFA experiment – supplementary simulation data

Supporting particle-in-cell (PIC) simulations were conducted using the code OSIRIS [1], in order to offer further insights into the differences observed in the experiment between the self–ionized and pre–ionized PWFA scenarios. These simulations start right before the second stage, where the main electron beam is assumed to be at waist. The beam is initialized according to a Gaussian distribution in phase-space with no correlation between the different coordinates. The total (FWHM) charge of the beam is 158 pC (120 pC), the average (rms) energy is 260 MeV (10 MeV), emulating what was measured in the experiment with the second gas jet turned off. Other parameters of the beam are 5 µm transverse spot size (rms), 18 fs duration (FWHM), and a peak current of 8 kA. Finally, the normalized emittance is equal to 5 µm rad in both transverse planes. The hydrogen gas profile in the simulation consists of a Gaussian up-ramp with a total length of 0.9 mm, followed by a constant plateau with a gas density of $2 \times 10^{18}\,\mathrm{cm}^{-3}$. For the self–ionized case only the ramp is initialized pre–ionized (due to the presence of the laser, the hydrogen is assumed fully ionized before the foil, but no laser-driven plasma waves have been simulated here), while the plateau section emulates a simple neutral molecular hydrogen species with two ionization levels, simulated according to the ADK tunnel ionization model [2]. In the pre–ionized case, also the plateau is initialized fully ionized. In this simulation series, the effect of the passage of the electron beam through the steel foil is neglected.
Supplementary Fig. 4 shows the simulation results on the drive beam deceleration after interaction with the PWFA stage for both the self–ionized and pre–ionized case. The electron density snapshots shown in Fig. 1b-c in the main manuscript correspond to 0.8 mm of propagation (at the center of the PWFA stage). In qualitative agreement with experimental observations, the pre-ionized case leads to a significant increase in energy spread, according to a decrease in the associated spectral charge density.

Supplementary Note 4: Drive-witness bunch pair LPWFA experiment – supplementary discussion

The externally seeded hybrid LWFA-PWFA experiment using driver-witness bunches was carried out with the ATLAS Ti:Sapphire laser system, situated at the Laboratory for Extreme Photonics (LEX) at Ludwig-Maximilians-Universität (LMU) München. It delivered 2.5 J pulses of ∼28 fs FWHM duration (∼80 TW) at 800 nm central wavelength. The pulses were focused on the first gas target in a $F/25$ geometry to a peak intensity of $6.8 \pm 0.5 \times 10^{18}\,\mathrm{W\,cm^{-2}}$.
In this experiment, two supersonic de Laval gas nozzles were used. The outlet diameters and Mach numbers of the first (LWFA) and second (PWFA) nozzle was 5 mm, $M = 6.35$ and 1 mm, $M = 5$, respectively, with a vacuum-gap of 5.5–6 mm. A shock-front injector was used to trigger injection of an electron bunch into the first and second wake bucket in the LWFA stage. The shock was created by the edge of a silicon wafer partially obstructing the gas flow. Supplementary Figure 5 shows the interferometrically measured electron density profile along the interaction axis. The electron energy beyond a lower cut-off of 25 MeV was measured using a 80 cm-long, 0.85 T permanent dipole magnet spectrometer.
The shock-front injection into the first and second bucket creates a driver and witness beam. During propagation in the second gas jet, the driver excites a beam-driven plasma wave which is captured transversely utilizing an ultrafast shadowgraphic imaging technique, see Supplementary Figure 6, similar to Gilljohann et al., PRX 9, 011046 (2019). In order to enhance the image contrast, this imaging was done by operating the PWFA stage at a higher plasma density (about 10 times) compared to the PWFA energy gain demonstration experiment. This plasma wave detected at higher density is proof for the existence of a more strongly driven wave at lower density due to a higher beam-to-background density ratio. The drive-witness bunch pair parameters, derived from a Gaussian fit to both energy peaks in each single–shot spectrum are given in Supplementary Table 3 and both the averaged experimental spectra as well as the fitted Gaussian–distributions are visualized in Supplementary Figure 7. Note that data for higher pressure shown in Fig. 4 of the main manuscript are not included in neither the table nor the plot, as most of the merging spectra cannot be unambiguously divided into a driver and a witness part.
The representative single-shots included in Fig. 4 of the main manuscript are shown as 2-D raw images for the LWFA reference in Supplementary Fig. 8a and the LPWFA scenario in Supplementary Fig. 8b. Note that, in order to accurately measure the electron bunch energy on every single shot, we used a pointing screen to determine the precise

position of the electron beam at the spectrometer entrance. Scattering in that screen broadens both the divergence and the energy spread, but due to the shape of the spectrometer dispersion curve has negligible influence on position of the peak energy. Therefore, the divergences of the driver and witness cannot be deduced from the spectrometer screen. The divergence integrated over the full spectrum as recorded by the pointing screen can still be analyzed by fitting a double-Gaussian to the beam profile to distinguish the well–collimated high–energy electrons dominated by the drive bunch from a broader low-energy background. The average over all shots without the second jet on (see blue curve in Fig. 4a) yields a native drive beam divergence of 1 mrad FWHM. Due to their origin from shock-front injection into two subsequent buckets they are expected to be separated by approximately one plasma wavelength (17 µm at $n_e = 3.7 \times 10^{18}\,\mathrm{cm}^{-3}$). This assumption is supported by a quasi-3D PIC simulation using the code FBPIC [3] for the parameters of the experiment. The result is shown in Supplementary Fig. 9. The top frame shows the electron density distribution at the end of the plateau region of the first gas jet. The drive laser pulse is located at a position of $\zeta = 0$, and the first and second bunches are following 12.5 µm and 30.5 µm behind, respectively. The bottom frame shows the situation at the position of the strongest acceleration field in the second jet. The drive bunch creates a plasma blowout, and the second bunch sits in an accelerating phase with a peak local field of approx. $30\,\mathrm{GV\,m^{-1}}$, consistent with the observed energy gain in the experiment.

Supplementary References

1. R. A. Fonseca, L. O. Silva, F. S. Tsung, V. K. Decyk, W. Lu, C. Ren, W. B. Mori, S. Deng, S. Lee, T. Katsouleas, and J. C. Adam, Notes Comp. Sci. **2331**, 342 (2002).

2. M. V. Ammosov, N. B. Delone, and V. P. Krainov, Sov. Phys. JETP **64**, 1191 (1986).

3. R. Lehe, M. Kirchen, I. A. Andriyash, B. B. Godfrey, and J.-L. Vay, Comput. Phys. Commun. **203**, 66 (2016).
